# Emergency Navigation without an Infrastructure

**DOI:** 10.3390/s140815142

**Published:** 2014-08-18

**Authors:** Erol Gelenbe, Huibo Bi

**Affiliations:** Intelligent Systems and Networks Group, Department of Electrical and Electronic Engineering, Imperial College London, London SW7 2BT, UK; E-Mail: huibo.bi12@imperial.ac.uk

**Keywords:** coordinated emergency navigation, infrastructure-less, cloud computing, *ad hoc* cognitive packet networks

## Abstract

Emergency navigation systems for buildings and other built environments, such as sport arenas or shopping centres, typically rely on simple sensor networks to detect emergencies and, then, provide automatic signs to direct the evacuees. The major drawbacks of such static wireless sensor network (WSN)-based emergency navigation systems are the very limited computing capacity, which makes adaptivity very difficult, and the restricted battery power, due to the low cost of sensor nodes for unattended operation. If static wireless sensor networks and cloud-computing can be integrated, then intensive computations that are needed to determine optimal evacuation routes in the presence of time-varying hazards can be offloaded to the cloud, but the disadvantages of limited battery life-time at the client side, as well as the high likelihood of system malfunction during an emergency still remain. By making use of the powerful sensing ability of smart phones, which are increasingly ubiquitous, this paper presents a cloud-enabled indoor emergency navigation framework to direct evacuees in a coordinated fashion and to improve the reliability and resilience for both communication and localization. By combining social potential fields (SPF) and a cognitive packet network (CPN)-based algorithm, evacuees are guided to exits in dynamic loose clusters. Rather than relying on a conventional telecommunications infrastructure, we suggest an *ad hoc* cognitive packet network (AHCPN)-based protocol to adaptively search optimal communication routes between portable devices and the network egress nodes that provide access to cloud servers, in a manner that spares the remaining battery power of smart phones and minimizes the time latency. Experimental results through detailed simulations indicate that smart human motion and smart network management can increase the survival rate of evacuees and reduce the number of drained smart phones in an evacuation process.

## 1. Introduction

The large amount of multi-domain sensory information generated by increasingly advanced sensor networks can aggravate the burden of emergency navigation systems, which demand a time-critical response with respect to data collection, interpretation and transmission. Traditional WSN-based emergency management systems, which consist of functionality-identical sensors, have difficulty in offering optimal evacuations in a timely manner in the presence of time-varying hazards, due to the limitation in processing capability, battery power and communication speed [1]. Although the evolution from homogeneous architectures to heterogeneous architectures with functionally-separated nodes makes WSN-based systems more energy-efficient and fault-tolerant, this structure still suffers from resource restriction problems due to cost and unattended operation during lifetime.

Owing to the high processing power, large storage, low risk level and high interoperability, cloud computing has become a dominating technology and has the potential to revolutionise the emergency management landscape. Accompanying this tendency, a new interest has been aroused to consider smart phones as simple clients for the back-end cloud due to their large-scale adoption and sensing abilities [2,3]. Compared with WSN-based counterparts, smart phone-aided, cloud-based emergency response systems are more flexible due to the elimination of both the extensive time to setup a permanent WSN and the routine maintenance, such as battery changes. Furthermore, owing to the enormous processing power, cloud computing can be beneficial in providing better quality of information (Qol) [4] and making more sophisticated decisions. Additionally, cloud-based emergency navigation systems can decrease the likelihood of being affected by the ongoing hazard and reduce energy consumption of thin clients by offloading computations to the remote cloud.

However, state-of-the-art cloud-based emergency navigation systems, which are based on the client-server model, still face many challenges. For instance, most systems adopt optimal path-finding algorithms that demand time-consuming full graph searches and information synchronization processes, especially when a large-scale environment is involved. Meanwhile, the gathering and disseminating of a large volume and a wide variety of sensory information, such as multi-media geographical data, can deplete client devices quickly. Moreover, clients in existing systems are presumed to be trusted and cooperative. Hence, misbehaving clients or incorrect reading of sensors can have a significant impact on the system performance.

Thus, in this paper, we propose a novel cloud-based emergency navigation system that borrows the concept of a cognitive packet network (CPN) [5] and social potential fields [6] to evacuate civilians in built environments. As a software-defined network, CPN can be completely implemented in software and, therefore, is easy to deploy on cloud servers as virtual “CPN nodes” in a distributed manner. In addition, CPN can achieve fast adjustments, because each CPN node operates as a sub-system to detect the environment, and no synchronization procedure is required. In this framework, smart phones are used as clients to connect to the access point(s) of cloud servers that carry out the intensive computations. Evacuees are directed to exits with a cooperative strategy to improve the elasticity in communication and localization, as well as to increase the possibility for a civilian to obtain assistance when at risk. A power-aware QoS metric [7], which is based on the *ad hoc* cognitive packet network (AHCPN) protocol [8] that uses an energy-aware sensible routing technique [9], is also presented to prolong the lifetime of smart handsets. One advantage of the AHCPN protocol is that it can locate malicious users readily, as detailed path information is contained in the route-search packets. When an emergency happens, we assume that a few cloud access points can be rapidly deployed between the emergency locations and the cloud and that the evacuees themselves are only equipped with smart phones that constitute an energy-efficient *ad hoc* network between each other, so as to reach the access points for two-way communication.

Since mathematical models [10] cannot handle the full complexity that is encountered during an emergency and evacuation drills are high-priced and time-consuming, agent-based simulation methods [11–13] are indeed the tools of choice to study such systems. Hence, in this work, the proposed algorithms are evaluated in a simulated built environment where a fast-spreading fire breaks out.

The remainder of the paper is organized as follows: Section 2 presents the related studies of cloud-enabled emergency navigation systems and coordinated behaviour-involved algorithms in emergency evacuation. In Section 3, we recall the concept of the cognitive packet network and then describe the system architecture and related algorithms in Section 4. The simulation model and assumptions are introduced in Section 5, and results are presented in Section 6. Finally, we draw conclusions and present future work in Section 7.

## 2. Related Work

### 2.1. Cloud-Based Emergency Management Systems

Cloud computing provides a service-oriented architecture that harnesses the computing capability of massive inter-networked computers. Since the concept was explored in the 1960s, the scope of cloud computing has grown tremendously over the last few decades and has motivated considerable research in the field of emergency management. For example, [14] proposes a community-based cloud-enabled emergency management system that leverages existing social network sites (SNS) to collect and disseminate emergent information. This platform consists of a information repository layer and a social networking layer, and a semantics-based query is used to overcome heterogeneous data from diverse organizations. In [15], an intelligent transportation system (ITS) is presented with the aid of vehicular *ad hoc* networks (VANETs) and cloud computing. Different computational models are available in the cloud to generate the optimum strategy for diverse situations. Simulations are performed in the transportation network of a city where the Lighthill-Whitham-Richards (LWR) model is used to mimic the traffic. Results indicate that this large-scale emergency response system, which can collect and propagate information through different communication modes, overcomes the traditional analogues using media. Because of the handicaps, such as limited battery power and computing speed in the hybrid fire evacuation system (HBFES) [16], which uses mobile phones and radio frequency identification (RFID) to locate evacuees and calculate evacuation routes, in [17], this system is extended by employing an external cloud server to perform computations. Due to the limitations of common indoor positioning technologies and the unavailability of GPS, in [18], a smart phone-assisted system to locate evacuees with pedometry-based localization and image-based positioning is suggested. The cloud-based server can obtain the position of individuals by matching the image snapshots uploaded by evacuees and then provide uncrowded routes for users. However, current cloud-enabled emergency response systems with the aid of portable devices do not consider the impact of significant energy utilisation in the client side during the communication process. On the other hand, smart phones using the Universal Mobile Telecommunications System (UMTS) protocol require base stations and a substantial infrastructure, which may be destroyed or overloaded during an emergency. Furthermore, UMTS systems themselves can easily come under attack [19,20], including by various forms of denial of service attacks [21].

### 2.2. Coordination Algorithms for Evacuation

Much research has demonstrated that cooperative strategies have a significant positive influence on multi-agent systems, and related studies have emerged for decades as organizational paradigms in agent-based structures [22] and autonomous search by large-scale robotic systems [6,23]. However, most related research on emergency evacuation focuses on developing diverse models [24] to simulate the crowd or coordinated behaviours, such as kin behaviour [25] in emergencies.

“Social potential fields”, which were initially used for coordinating robots in a very large-scale robotic system [6], have been employed for emergency navigation in [26]. A self-organizing sensor network is established with “artificial potential fields” where artificial forces exist. Exits and dangerous zones generate attractive forces and repulsive forces, respectively, and evacuees move under the actuation of the resultant force. However, the original SPF considers the interactions between robots to avoid congestion, while the artificial potential fields in [26] does not take influences among evacuees into account.

Emergency evacuation often accompanies the malfunction of communication infrastructures or sensors, which can be caused by an on-going hazard or malicious attacks. The failure of the communication infrastructure can render a centralized system invalid, while the malfunction of sensors will affect the QoI and accuracy of localization. To solve the above problems, some coordinated behaviour-involved algorithms, such as opportunistic communications and delay tolerant networks, have been investigated to increase the resilience of emergency management systems [27–29]. For instance, [30–32] have proposed a resilient emergency support system (ESS) with the aid of opportunistic communications [33]. This system consists of sensor nodes (SNs) and communication nodes (CNs). SNs can detect the hazard in its vicinity and inform the evacuees passing by of the location, while CNs are portable devices that are taken by occupants. The civilians initially wander in the environment and can only inform the civilians about the hazard within its communication range. Each civilian calculates the shortest path to the exits with Dijkstra's algorithm. However, this work concentrates on studying the impact of “passive” cooperation among evacuees, who exchange emergent messages with other civilians, and no active mechanism is used to improve the information dissemination efficiency.

## 3. Cognitive Packet Network

Searching physical routes for evacuees or communication routes for emergent messages can be modelled as nonlinear combinatorial optimisation problems to search optimal paths, while minimizing desired cost functions [34]. Due to the fact that classical optimization algorithms can be easily trapped in local minima [35] and exact solutions can be computationally expensive and time consuming for NP-hard problems [36], in this paper, we use the concept of the cognitive packet network, which is a meta-heuristic inspired by natural intelligence, to emergency planning problems.

The cognitive packet network (CPN) introduced in [37–39] was originally proposed for large-scale and fast-changing packet networks and can provide QoS-driven routing to satisfy diverse end users in multimedia networks. By employing smart packets (SPs), CPN can discover optimal routes rapidly and heuristically and realise continuous self-improvement. Contrary to conventional routing protocols, in the CPN, intelligence is realised by using random neural networks (RNNs) [40–43], which is a mathematical model of natural mammalian neuronal networks and is constructed into SP search. Hence, SPs can discover optimised routes with their predefined goals and improve QoS by learning from their own investigations and experience from other packets.

CPN has been applied to many domains, including emergency navigation [44,45] and emergency resource allocation [35,46–48]. As the world's first software-defined network (SDN), it can be installed on computers seamlessly [49]. In the proposed system, the CPN-based algorithm is deployed in the back-end cloud, and each node in the CPN represents a realistic location where evacuees can identify their locations by uploading visual snapshots and querying the cloud. Each node is composed of a recurrent RNN to determine the direction of SPs and a mailbox (MB). Each neuron in the RNN represents a neighbour node, and the neighbour node associated with the highest excitation probability indicates the most beneficial forwarding direction of SPs. The notation in Table 1 is the same as that used in the original RNN model [40]. The mailbox maintains a routing list to store the paths discovered by SPs and sensory data uploaded by smartphones. Consequently, the original WSN-based CPN algorithm in [44] is virtualised in the cloud. CPN contains three types of packets: smart packets (SPs), acknowledgements (ACKs) and dumb packets (DPs). SPs gather information from the traversed nodes and search paths to destinations with respect to predefined QoS goals. They can either choose the neighbour node decided by random neural networks as the next hop or drift randomly to explore and update routes. When an SP reaches the destination, an ACK, which reserves the discovered path, and collected data will be generated and sent back to the source node through the reverse path. When an ACK reaches a node, it will update the MB and train the random neural network by performing reinforcement learning (RL), as shown in Pseudocode 1. DPs are the packets that actually carry the payloads. they always choose the top-ranked path in the routing list as the next hop. In the context of emergency evacuation, evacuees are considered as DPs.

AHCPN [8] is a variant of the original CPN and is used in the high dynamic mobile *ad hoc* networks (MANETs) in which the position of users varies continuously. Since CPN can allocate resources in proportion to the probability of finding an optimal route there, it can significantly replace broadcast transmissions in traditional routing protocols with unicasts. Because each smart phone can communicate with the cloud via 3G directly, hence each smart phone can be considered as a local “exit”, and SPs need to decide when to stop searching among mobile phones and transmit to the access node that connects to the remote cloud. Benefiting from their inherent intelligence, SPs can self-determine to transmit to the cloud when any of the two following conditions is fulfilled:

- If all of the neighbour portable devices of the current smart handset in the near-field communication range have already been visited by checking the cognitive map of SPs.
- If the predicted path availability (calculated by Formula 3) of conveying a payload from the current discovered path is larger than the estimated path availability of transmitting directly from the source smart phone.

Furthermore, emergencies caused by terrorist attacks can also be accompanied with network attacks, such as node capture or denial of sleep in the client side and denial of service in the server side [50]. CPN can naturally benefit the process of defending malicious acts, because SPs and ACKs store all of the intermediate nodes from the source to the destination. Therefore, the potential malicious nodes can be located easily. All of the nodes between a malicious origin and the target node can drop attacking traffic to avoid useless occupancy of bandwidth. Meanwhile, as CPN is a QoS-driven protocol, packets can determine the validity by QoS, and smart phones can allocate different bandwidth for flows different QoS.


**Pseudocode 1** The training process of RNN.
**Input:** Path information brought back by an ACK**Output:** The excitation probability of neurons in the RNN1:When an ACK reaches a node, extract gathered path information from the ACK2:Calculate the path cost G based on the collected information and the associated goal function3:Compute the reward 
$R_{l}=\frac{1}{G}$4:Calculate the decision threshold *T_l_* = *aT_l_*_−1_ + (1−*a*) *R_i_*5:**for** each neuron *i* in the RNN **do**6: /* Compute the current total firing rate coming out of neuron *i* */7: 
$\begin{array}{cc} r\left(i\right)=\sum_{m=1}^{n}\left[w^{+}\left(i,m\right)+w^{-}\left(i,m\right)\right] & \left(m\neq i\right) \end{array}$8:**end for**9:**for** each neuron *i* in the RNN, except neuron *j*
**do**10: /* Compare the *R_l_* with the previous threshold *T_l_*_−1_ and update weights of neurons */11: **if**
*R_l_* ≥ *T_l_*_−1_
**then**12:  
$w^{+}(i,j)\leftarrow w^{+}(i,j)+\left|R_{l}-T_{l-1}\right|$13:  
$w^{-}(i,k)\leftarrow w^{-}(i,k)+\frac{\left|R_{l}-T_{l-1}\right|}{n-2}$14: **else**15:  
$w^{-}(i,k)\leftarrow w^{-}(i,k)+\left|R_{l}-T_{l-1}\right|$16:  
$w^{-}(i,k)\leftarrow w^{-}(i,k)+\frac{\left|R_{l}-T_{l-1}\right|}{n-2}$17: **end if**18:**end for**19:**for** each neuron *i* in the RNN **do**20: /* Compute the updated total firing rate of neuron *i* */21: 
$\begin{array}{cc} r{\left(i\right)}^{*}=\sum_{m=1}^{n}\left[w^{+}\left(i,m\right)+w^{-}\left(i,m\right)\right] & \left(m\neq i\right) \end{array}$22:**end for**23:**for** each neuron *i* in the RNN **do**24: /* Normalize the weights of the neurons */25: **for** each linked neuron *m* of neuron *i* in the RNN **do**26:  
$w^{+}(i,m)\leftarrow w^{+}(i,m)*\frac{r\left(i\right)}{r{\left(i\right)}^{*}}$27:  
$w^{-}(i,m)\leftarrow w^{-}(i,m)*\frac{r\left(i\right)}{r{\left(i\right)}^{*}}$28: **end for**29:**end for**30:**for** each neuron *i* in the RNN **do**31: /* Calculate the excitation probability *q_i_* of the neuron *i* */32: 
$\begin{array}{cc} \lambda^{+}\left(i\right)=\sum_{m=1}^{n}q_{m}w^{+}\left(m,i\right)+\Lambda_{i} & \left(m\neq i\right) \end{array}$33: 
$\begin{array}{cc} \lambda^{-}\left(i\right)=\sum_{m=1}^{n}q_{m}w^{-}\left(m,i\right)+\lambda_{i} & \left(m\neq i\right) \end{array}$34: 
$q_{i}=\frac{\lambda^{+}\left(i\right)}{r\left(i\right)+\lambda^{-}\left(i\right)}$35:**end for**


## 4. The Cloud-Enabled Emergency Navigation System

**Figure 1.**
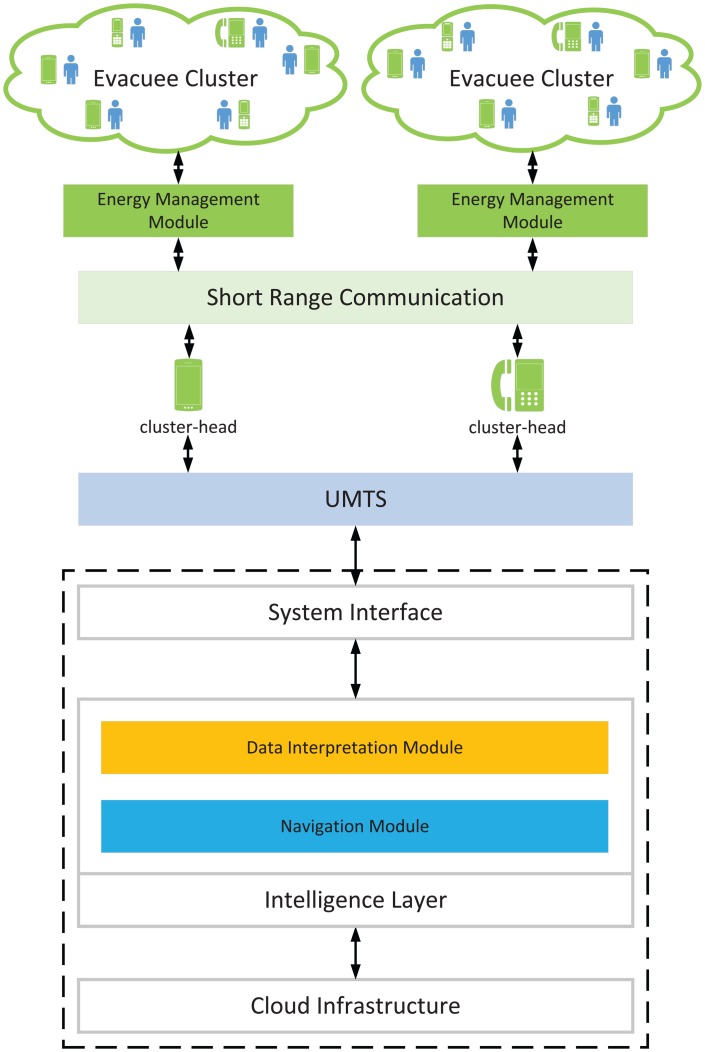
The architecture of the cloud-enabled system.

Figure 1 shows the architecture of the cloud-enabled system. The system is composed of four layers. The cloud infrastructure layer is a hardware layer that provides the platform for the deployment of the system. The intelligence layer comprises a data interpretation module and a navigation module. The data interpretation module aims to provide better QoI for decision making, while the navigation module is used to guide evacuees to exits in a cooperative manner. The system interface layer is responsible for information exchanges from diverse gateways, such as the Universal Mobile Telecommunications System (UMTS) or Internet. Civilians with smart phones form the user layer to collect sensory data and convey it to the cloud. An AHCPN-based energy management module is installed among the handsets to search routes to realise energy-sensitive communication between the cloud and handhold devices.

The main objectives of the system are to provide appropriate routes for evacuees in a real-time manner and to balance the energy dissipation of portable devices. When an evacuation process starts, hazard information will be gathered by individuals and then uploaded to the cloud. On the cloud side, the CPN algorithm with a time metric will be performed to search paths with the shortest “time” to exits. Furthermore, a smart group motion will be generated by the SPN algorithm, which employs attractive forces and repulsive forces to maintain adjacent evacuees as a loose group. The suggested directions will be brought back by ACKs and visualised on the screens of portable devices. The simplified workflow of the system is shown in Pseudocode 2, and a list of symbols used is summarised in Table 2. In the following subsections, details of the coordinated emergency navigation algorithm in the navigation module and the power-aware protocol in the energy management module are introduced.

### 4.1. Coordinated Emergency Navigation

As emergency navigation systems always perform in harsh physical environments, the likelihood of the malfunction of components is relatively high, and the coordinated behaviour of evacuees can facilitate building a fault-tolerant framework. For example, the battery power of a smart phone can be depleted during an evacuation process. In this case, a single evacuee can be trapped owing to being unaware of the physical location. Coalition in evacuation can avoid this by increasing the likelihood of following other evacuees in proximity to exits. Furthermore, emergency navigation systems involve many data flows for information acquirement and dissemination. Therefore, evacuating in a cooperative manner can realise energy efficiency by relaying data or providing alternatives, such as opportunistic communication, when the main communication mode fails. Another advantage of evacuating in teams is to provide better QoI by merging the overlapping information. Hence, in this section, we leverage the SPF to cluster evacuees into groups during an evacuation.


**Pseudocode 2** The workflow of the system.
1:**while** an evacuee reaches a landmark **do**2: Collect *in situ* hazard information with smart phone's built-in camera3: Sense other smart phones in the near-field communication range4: **if** the neighbouring smart phones has changed **then**5:  Reset the RNN structure in the AHCPN-based energy management module6:  Set *RNN_new_* → *true*7: **end if**8: /* Emit *N_m_* SPs to search energy-efficient communication routes */9: **while**
*N_c_ < N_m_*
**do**10:  **while**
*H_c_ < H_m_*
**do**11:   Generate a random value R between 0 and 112:   **if**
*R*< *V_Drift_* & ¬*RN N_new_*
**then**13:    Choose the neighbour associated with the most excited neuron as the next hop14:   **else**15:    Choose a random neighbour as the next hop16:   **end if**17:   /* When a SP reaches a new smart phone */18:   **if**
$P_{\text{Bluetooth}+3G}^{i}\geq P_{3G}^{i}$
**then**19:    Stop searching and transmit the SP to the cloud access point via 3G20:   **else**21:    Check the current discovered route in the cognitive map of the SP22:    **if** all of the neighbours of the current smart phone have been visited **then**23:     Stop searching and convey the SP to the cloud access point via 3G24:    **else**25:     Add the current smart phone to the cognitive map of the SP26:    **end if**27:   **end if**28:   **end while**29:   Generate an ACK to bring back gathered delay and energy information30:  **end while**31: Choose the top ranked path P in the routing list to send the snapshot32: Cloud severs employ image processing algorithms to identify the location of the evacuee and the hazard intensity33: Update the related hazard situation in the building graph34: Generate a random value *R* between 0 and 135: **if**
*R* > *V_SPN_*
**then**36: Choose the decision made by the CPN-based algorithm37: **else**38: Choose the decision made by the SPN-based algorithm39: **end if**40: Send the decision back to the evacuee through the reverse of path *P*41:**end while**


The initial SPF algorithm [51] consists of global control forces and local control forces. The global control forces coordinate the individuals and determine the distribution of the individuals, while the local control forces dominate the personal behaviours. In our treatment, we replace the local control forces with the CPN-based algorithm to control the individual behaviour of evacuees and use the global forces to regulate the intra-group behaviours. Details of the CPN-based emergency evacuation algorithm can be seen in [44] and [45]. The force between two evacuees can be calculated from the following equation:

(1) $$f\left(r\right)=-\frac{c_{1}}{r^{\sigma }1}+\frac{c_{2}}{r^{\sigma }2}$$

where c_1_, c_2_, σ_1_ and σ_2_ are dynamic parameters and *r* represents the physical distance between two civilians. Term $-\frac{c_{1}}{r^{\sigma }1}$ represents the repulsive force, while $\frac{c_{2}}{r^{\sigma }2}$ depicts the attractive force. We assume that each civilian can only be affected by other civilians within 20 meters. If the distance between two civilians is smaller than seven meters, a repulsive force will be generated; otherwise, an attractive force will be produced. To achieve this, we set c_1_ to 20, c_2_ to 15, σ_1_ to 0.9478 and σ_2_ to 0.8, respectively. This ensures that the civilians will evacuate in loose groups without increasing the level of congestion.

To combine SPF and the CPN-based algorithm, various schemes can be employed. For instance, the resultant direction can be calculated by the weighted sum model in which the two elements are assigned with different weights. Another simple scheme is to randomly choose either the decision of SPF or CPN as the next decision at a time. The latter scheme is employed in this system: when an SPF-related decision is chosen, we adopt the associated neighbour node that has the most matched direction with the resultant force as the next hop.

### 4.2. Power-Aware Protocol

As the front-end component of the framework, smart phones, which can offer diverse services, high data rate connectivity and sensing capabilities, play a vital role in the cloud-enabled system. However, due to the limitation on battery size and the efficiency of chemical reactions of the widely-used lithium-ion batteries [52], the capacity of batteries becomes a bottleneck in the operational time of the cloud-based system. In fact, the development of battery capacity does not keep pace with the computational complexity: the computational complexity is roughly doubled every two year with respect to Moore's law, while the battery capacity is doubled every decade [53].

Emergency evacuation is an energy-hungry process due to the large amount of information exchange involved. Experiments in [54] show that the energy consumption of air interfaces, such as Wi-Fi and GPRS, far exceed the power utilization of the RAM and CPU. Furthermore, the energy consumption for different communication modes varies. For instance, Bluetooth consumes much less energy than 3G mode during data sending and receiving [53]. Because the remaining battery power of portable devices is different when an emergency event occurs and the power utilisation rate of different handsets varies, it is not an optimal strategy for each smart phone to exchange information with the cloud through 3G directly. In this section, we make use of the cluster behaviours proposed in the aforementioned section to employ short-range communications to relay sensory information and, ultimately, upload the data from a certain number of mobile devices through 3G. Similarly, decisions from the cloud are not conveyed to each evacuee directly, but are transmitted to a certain evacuee and then distributed within the group using short-range communication technologies.

To realise energy efficiency and to maximise the overall average battery lifetime of smart phones, we employ an AHCPN-based algorithm to optimise the routes for the information flows. The AHCPN-based algorithm is deployed on the smart phones to search power-saving paths for conveying sensory data to the cloud. The QoS criterion we used is inspired by the energy-aware metric in [8]. It is a compound metric, which involves battery power and latency. Here, we also employ the “path availability” notion and construct a similar metric.

(2) $$G_{\text{ed}}=\alpha \prod_{i=0}^{n-1}P_{a}\left(\pi \left(i\right),\pi \left(i+1\right)\right)\left\{\sum_{i=0}^{n-1}D\left(\pi \left(i\right),\pi \left(i+1\right)\right)\right\}$$

where *π* represents a particular path, *n* is the number of nodes on the path *π* and *π*(*i*) is the i-th node on the path *π*. Hence, *π*(0) is the source node, while *π*(*n*) is the destination node (the cloud access point). Term *P_a_*(*π*(*i*),*π*(*i* + 1)) is the availability of the edge between *π*(*i*) and *π*(*i* + 1). *D*(*π*(*i*),*π*(*i* + 1)) is the delay cost for a packet to transmit from *π*(*i*) to *π*(*i* + 1). Term *α* is a constant that coordinates the relation between the path availability and delay. Because the average latency for uploading a photo is less than 0.1 s and the path availability is normally slightly larger than one (due to the fact that the remaining battery power is usually much larger than the energy consumption of uploading a snapshot), term *a* is set to 100 to amplify the result of the goal function and to ensure the effective training process of random neural networks.

The path availability is affected by the remaining battery power of a smart phone and the estimated power consumption of transmitting a piece of information:

(3) $$P_{a}\left(\pi \left(i\right),\pi \left(i+1\right)\right)=\frac{B_{i}^{C}}{B_{i}^{C}-B_{i}^{U}}$$

where $B_{i}^{C}$ represents the current remaining battery power of a node (smart phone) *π*(*i*) and $B_{i}^{U}$ depicts the power utilisation at node *π*(*i*) to convey a certain piece of information. It is estimated by the number of packets that will be transmitted. If the potential power utilisation is larger than the remaining battery, then this node is excluded. The path availability of a whole route is the product of the availability of edges on it.

## 5. Simulation Model and Assumptions

**Figure 2.**
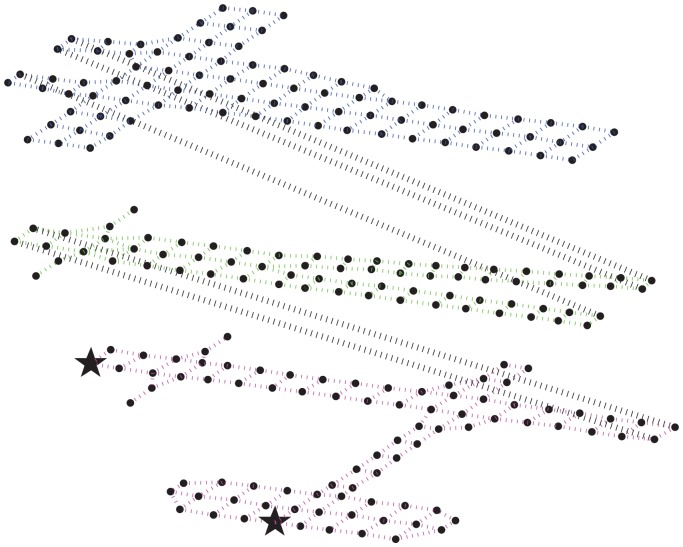
Graph representation of the building model. The two black stars on the ground floor mark the position of the building's exits.

We use an agent-based discrete-event simulator, namely the Distributed Building Evacuation Simulator (DBES), to evaluate the effectiveness of the novel navigation algorithm, as well as the AHCPN-based energy-efficient protocol in a fire-related emergency evacuation. The physical area is hypothesised as a graph: vertices are positions with landmarks by which evacuees can easily identify their locations by uploading snapshots and querying the server; edges are physical links by which evacuees can move inside the building. The graph can be downloaded from the cloud when a hazard occurs. The building model we employed is a large canary wharf shopping mall with relatively wide paths for evacuees to move in clusters. The shopping mall is a three-story building with two exits on the ground floor, as shown in Figure 2. Initially, evacuees are randomly scattered in the building, and they are assumed to be equipped with smart phones with random remaining battery power. When the battery power of smart phone is depleted, the evacuee will wander or follow other evacuees in the line of sight to the exits.

We assume that no related sensors are pre-installed in the built environment. Hence, hazards can only be discovered by residents in the building. When an evacuee discovers a hazard, he/she will inform the server by using the smart phone, and then, the server will alarm all of the related civilians. Civilians can locate themselves by taking image snapshots and matching them with pre-known landmarks, which are stored in cloud severs. The photos are taken by built-in cameras on the mobile phones and then uploaded to the cloud. The image-based localization algorithms [18] are performed in cloud severs. The spreading of the hazard is reported by evacuees and predicted by hazard models in the cloud.

In the simulation, the indoor Wi-Fi access points are presumed to have malfunctioned due to hazards or the outage of the power supply. Hence, the portable devices can only communicate with the cloud severs through UMTS. To maintain the 3G communication between the cloud and smart handsets, we hypothesise that during an emergency, a few cloud access points can be quickly deployed in the vicinity of the building. We also assume that smart phones provide the Bluetooth communication mode to conduct short-range communications. The size of the image taken by smart phones is set to 100 kilobytes, and the header of the AHCPN packets is 12 bytes [8]. The smart phone identification uses a IP address space which is four bytes. The initial battery power of smart phones yields a normal distribution, and the battery capacity of smart phones is set to 16,000 joules.

The energy consumption model of smart phones is based on the previous literature [54–57]. We assume that smart phones have three states: suspended state, idle states and high-power state. When in a suspended state, the application processor of smart handsets is idle and the communications processor performs a low level of activity [54]. When in an idle case, the smart phone is fully awake, but no application is active. Finally, the high-power case is the state when the portable device interacts with the cloud. The power utilisation of different states with regard to the elapsed time can be seen in Table 3, which is based on the average system power of specific smart phones for a selection of benchmarks in [55].

The energy utilization of different communication modes with respect to the amount of transferred data, as well as the associated transmission rate are shown in Table 4. This model is based on the energy consumption ratios presented in [57] and the measurement study of energy consumption over 3G and Wi-Fi in [56].

## 6. Results and Discussion

To evaluate the navigation algorithm that combines CPN and SPF (CPN&SPF) as well as the AHCPN-based energy-efficient protocol, we design an experiment that involves four scenarios with 30, 60, 90, 120 evacuees, respectively. Dijkstra's shortest path algorithm and CPN-based algorithm with a time metric (CPNST) [45] are performed for comparison purpose. Evacuees can communicate with the cloud through UMTS directly or use Bluetooth to relay data to other smart phones with a higher percentage of remaining energy and then transmitting to cloud severs through 3G.

**Figure 3.**
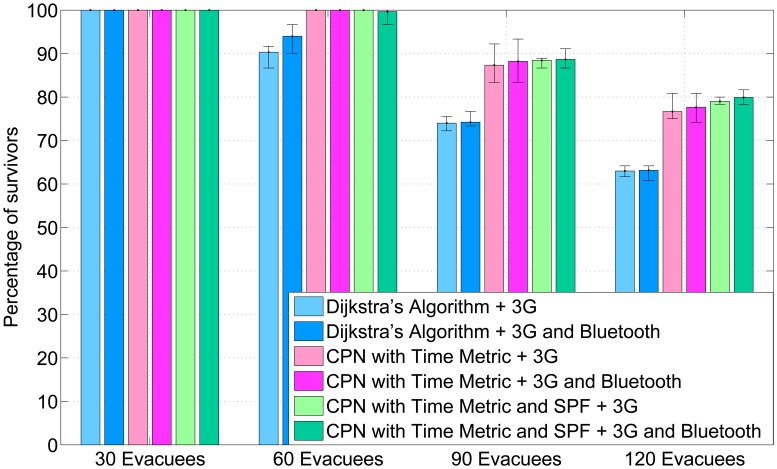
The percentage of survivors for each scenario. The results are the average of five randomized simulation runs, and error bars show the min/max result in any of the five simulation runs.

The percentage of survivors of five randomised experiments is shown in Figure 3. Owing to the benefit of wide paths in the shopping mall, all civilians are able to evacuate at a low occupancy rate (30 evacuees). However, in relatively high population densities, both CPNST and CPN&SPF result in more survivors than Dijkstra's shortest path algorithm. This is due to the congestion-ease mechanism in CPNST. Meanwhile, SPF can also ease congestion, because it can generate large repulsive forces for the evacuees in proximity. In comparison with CPNST, CPN&SPF performs slightly better in densely-populated scenarios (90 and 120 evacuees). This is because CPN&SPF organises evacuees as loose clusters and, therefore, increases the likelihood for an evacuee to upload data through other smart phones with more remaining battery power. As a result, the probability of being trapped due to the depletion of the smart phone is decreased. Furthermore, the coordinated behaviour increases the probability of an evacuee with a depleted smart phone to follow other evacuees rather than wandering in the building. This can be confirmed by the fact that the outcome of experiments using the AHCPN energy-efficient protocol is better than for the counterparts that use UMTS directly. The AHCPN protocol can reduce the likelihood of depleting a smart phone and, therefore, decreases the possibility of an evacuee being trapped in the built environment. The results we present are numerical in nature, while it would be interesting also to discuss detailed simulations in a virtual reality environment [58].

**Figure 4.**
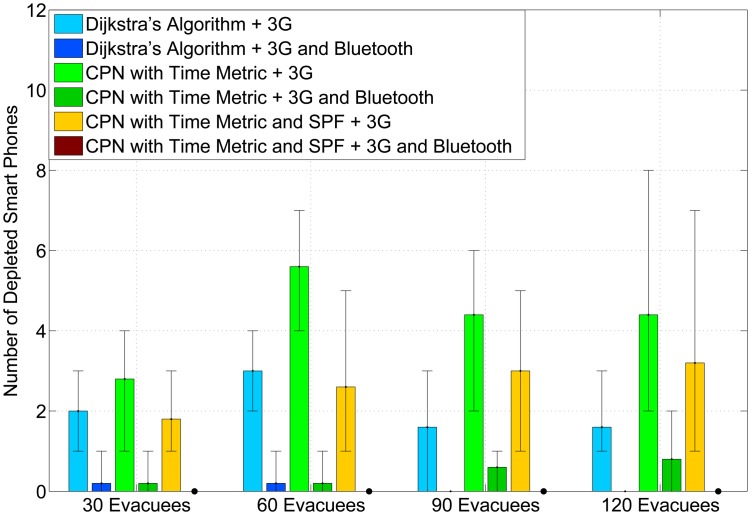
The number of drained smart phones for five iterations. The error bars represent the min/max values found in the five simulations. Please note that the bar for “CPN with Time Metric and SPF + 3G and Bluetooth” is not displayed, because there is no drained smart phone in this case.

Figure 4 presents the average number of drained smart phones in the experiments. When using 3G mode to communicate with the cloud, Dijkstra's algorithm achieves the least number of drained smart phones, especially at a high occupancy rate. This is because both the CPNST and CPN&SPF and do not follow the shortest path. Hence, evacuees will traverse more landmarks and upload more photos to the cloud. However, by employing the AHCPN protocol, the number of depleted smart phones decreases significantly for all three algorithms. Moreover, there is no drained smart phone when combining the CPN&SPF algorithm with the AHCPN protocol. This confirms that routing evacuees in loose groups can contribute to the power-balancing of smart phones.

**Figure 5.**
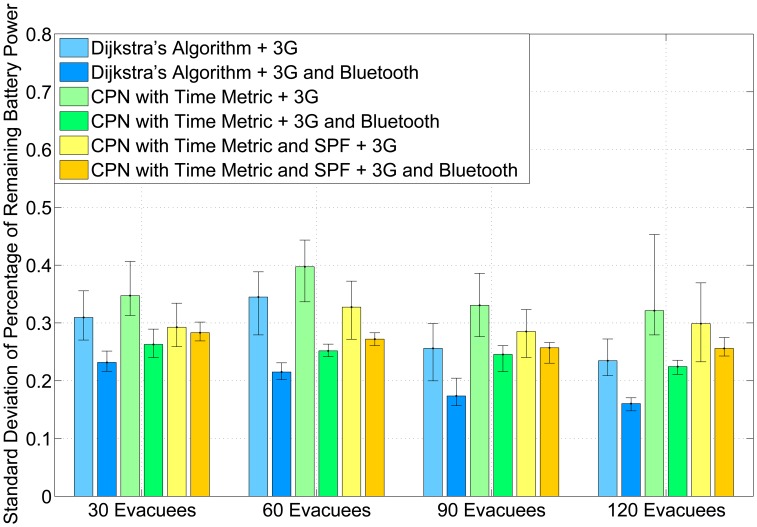
The average standard deviation of the percentage of remaining battery power of smart phones in five simulation runs. The error bars represent the min/max value of the standard deviation found in the five simulations.

Figure 5 shows the standard deviation of the remaining percentage of battery power for smart handsets. The remaining percentage of battery power *P_r_* in a smart handset is defined as follows:

(4) $$P_{r}=\frac{B_{r}}{B_{i}}$$

where *B_r_* is the remaining battery power of a handset and *B_i_* is the initial battery power of the smart phone. The result indicates that no matter which emergency navigation algorithm (Dijkstra's shortest path algorithm, CPNST or CPN&SPF) is used, the AHCPN-based protocol has a lower standard deviation of the remaining percentage of battery power in comparison with its counterpart, which transmits data to the cloud with 3G directly. Hence, although AHCPN may consume extra energy, because of sending smart packets periodically, it can balance the remaining battery power in portable devices and, therefore, prolong the lifetime of smart phones.

## 7. Conclusions and Future Work

In this paper, we propose an infrastructure-less, indoor emergency response system to evacuate civilians with the aid of smart handsets and cloud servers. A coordinated emergency navigation algorithm is proposed to guide evacuees in loose groups. The experimental results prove that the algorithm can increase the survival rate by reducing the number of drained smart phones in an evacuation process and raising the likelihood for an evacuee with a depleted mobile device to encounter other evacuees in the line of sight and follow them to egresses. Due to the considerable energy consumption between the cloud and smart phones during the communication processes, an AHCPN-based energy-efficient protocol is also presented to prolong the lifetime of smart handsets. Simulations indicate that the protocol can significantly decrease the number of drained smart phones in an evacuation process and balance the remaining battery power among portable devices. Future research will be directed to improve the flexibility of the system by leveraging the mobile agent technology, because current cloud-based emergency response systems that are based on the client-sever paradigm demand pre-installed services in participating devices [59]. Moreover, since mobile agents can migrate seamlessly through multiple clouds and different portable devices, a mobile agent-based emergency response system has the potential to reduce communication costs and ease network congestion in large-scale emergency evacuations by dynamically optimising the locations of mobile agents. We also think that cloud-based techniques can benefit systematic and repetitive issues, such as the clearance of improvised explosive devices (IOD) and land mines [60].

## Figures and Tables

**Table 1.** List of symbols used in Pseudocode 1. RNN, random neural network.

**Notation**	**Definition**
*a*	A constant between 0 and 1, typically close to 1
*n*	The number of neurons in a RNN
*j*	The neuron which is associated with the neighbour node that corresponds to the returned ACK
*k*	The other neurons in the RNN except the neuron *j*
*w*^+^(*i*,*j*)	Rate at which neuron *i* emits excitation spikes to neuron *j* when *i* is excited
*w*^−^(*i*,*j*)	Rate at which neuron *i* emits inhibition spikes to neuron *j* when *i* is excited
Λ*_i_*	Rate at which external excitation spikes arrive at neuron *i*
λ*_i_*	Rate at which external inhibition spikes arrive at neuron *i*
λ^+^(*i*)	Rate at which neuron *i* receives excitatory spikes
λ^−^(*i*)	Rate at which neuron *i* receives inhibitory spikes
*q_i_*	The excitation probability of neuron i
*R_l_*	*l*-th reward determined by a goal function
*T_l_*	*l*-th decision threshold that keeps track of the historical value of rewards

**Table 2.** List of symbols used in the Pseudocode 2. SP, smart packet.

**Notation**	**Definition**
*RNN_new_*	A Boolean value to indicate if a RNN is newly constructed or reset
*R*	A random generated value between 0 and 1
*N_m_*	The total number of SPs that can be emitted before sending a photo
*N_c_*	The number of SPs that has been emitted in a cycle
*H_m_*	The maximum number of hops a SP can traverse before being dropped
*H_c_*	Current traversed number of hops
*V_Drift_*	Probability to choose the neighbour associated with the most excited neuron as the next hop
*V_SPN_*	Probability to choose the SPN-based algorithm
$P_{\text{Bluetooth}+3G}^{i}$	Path availability (calculated by Formula 3) of transmitting a snapshot via the current route
$P_{3G}^{i}$	Path availability of conveying a snapshot via 3G mode

**Table 3.** The energy consumption of different states in joules with respect to the elapsed time in seconds. Term *x* represents the elapsed time, and *y* depicts the energy utilization on the smart phone side when performing the emergency navigation system.

**States**	**Energy Model**
Suspended	0.3394*x*
Idle	0.1049*x*
High power	0.1049*x* + *y*

**Table 4.** The energy consumption of smart phones in joules with regard to the transferred data *x* in byte and the related signal rate.

**Communication Mode**	**Download**	**Upload**	**Signal Rate**
3G	0.001224*x*	0.0003375*x*	2 Mb/s
Bluetooth	0.0001377*x*	0.00012012*x*	1 Mb/s
Wi-Fi	0.0003057*x*	0.0001586*x*	54 Mb/s
ZigBee	0.0002861*x*	0.0003164*x*	250 Kb/s
